# Multielement Composition and in Vitro Bioaccessibility in Brazilian Edible Floral Supplement Capsules Containing Dandelion and Hibiscus

**DOI:** 10.1007/s12011-026-05309-w

**Published:** 2026-09-03

**Authors:** Taís Lorena Santos Fiais, Jorge Machado Freitas Junior, Ygor Felipe Garcez Brito, Eliabe Sousa da Silva, Clícia Maria de Jesus Benevides, Thaís Luz de Souza, Jamille Santos Santana, Aníbal de Freitas Santos Júnior

**Affiliations:** 1 Department of Life Sciences, State University of Bahia (UNEB), Bahia, Salvador 41195-001 Brazil; 2 Department of Exact and Earth Sciences, State University of Bahia (UNEB), Bahia, Salvador 41195-001 Brazil; 3 Agricultural Technology Center of the State of Bahia (CETAB), Salvador, Bahia, 40170 110 Brazil

**Keywords:** Edible flowers, Multielement composition, INFOGEST 2.0 protocol, In vitro bioaccessibility, MIP OES

## Abstract

Dandelion (*Taraxacum officinale*) and hibiscus (*Hibiscus sabdariffa*) are notable unconventional edible flowers (UEFs) that have traditionally been used as food and in herbal preparations. This study examined the in vitro bioaccessibility content of six elements (Cr, Cu, Fe, Mn, Ni, and Zn) in edible flower capsules sold in Brazil. Acid digestion in a closed system and microwave-induced plasma optical emission spectrometry (MIP OES) were employed, as well as simulated gastrointestinal digestion according to the INFOGEST 2.0 in vitro protocol. Additionally, we examined the contribution of the in vitro bioaccessible fraction (FBio) in relation to the recommended daily intake (RDI). The concentrations (in µg g⁻^1^) of the target elements were as follows: Cr (< 0.46–2.82), Cu (< 0.48–11.45), Fe (159.44–1880.90), Mn (48.94–441.34), Ni (< 0.44–3.21), and Zn (< 0.55–30.98). The FBio of the evaluated elements ranged from 0.13% to 14.31%. Although Fe had high total levels, its bioaccessibility was limited, contributing only 0.13%. In contrast, Mn and Ni exhibited high gastrointestinal release, accounting for 12.13% and 14.31% of the FBio, respectively. Mn showed high gastrointestinal release, providing up to 209.3% of the RDI. This study is the first to specifically evaluate the bioaccessible trace element content of these edible flowers using the INFOGEST 2.0 in vitro protocol and MIP OES to assess microelements. Overall, the results highlight the importance of incorporating chemical composition and bioaccessibility data into the multi-element characterization of dandelion and hibiscus.

**Graphical Abstract:**

**Figure:**
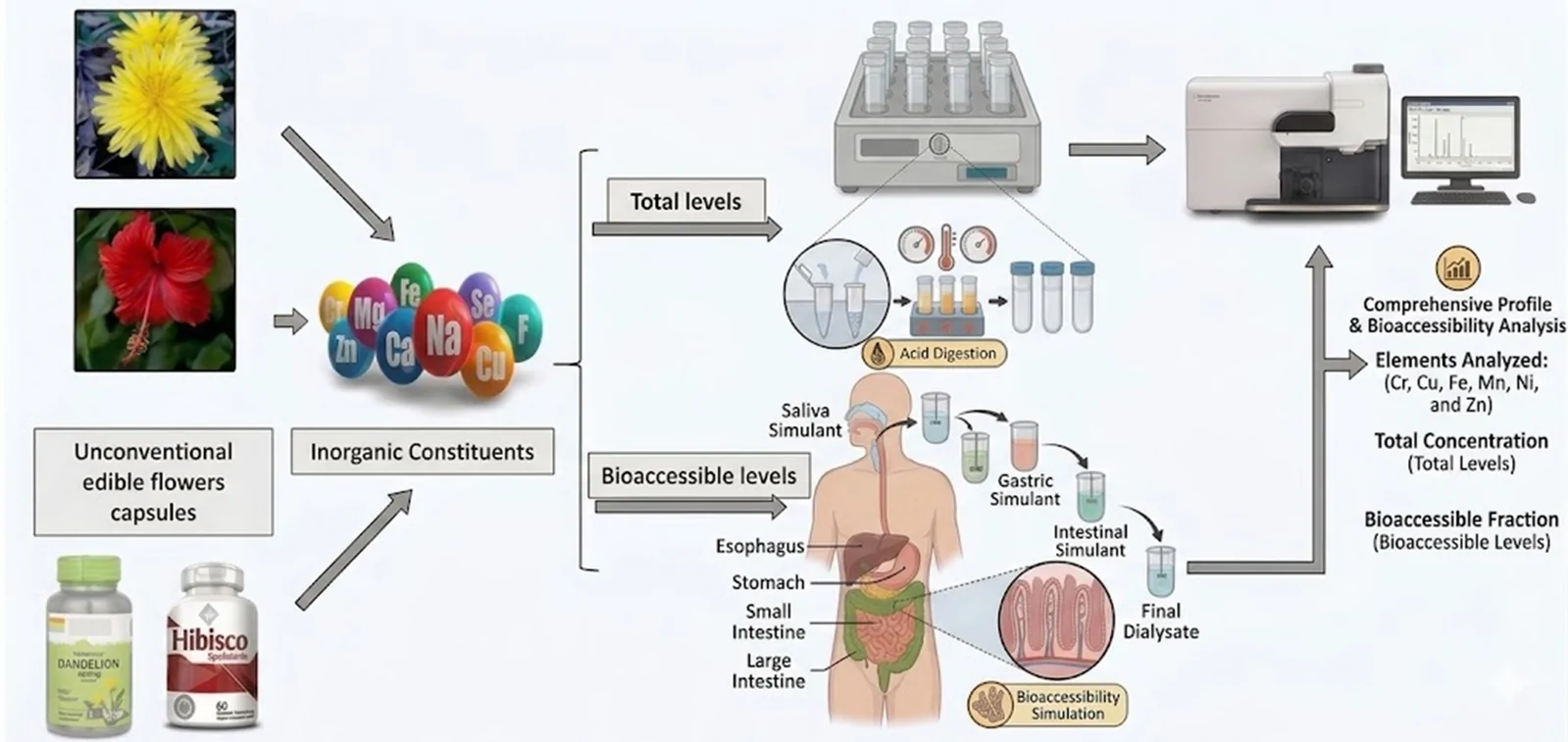

## Highlights

Combining MIP OES analysis with the INFOGEST 2.0 in vitro protocol provides insight into the mineral bioaccessibility of edible flower capsules sold in Brazil.

Mass balance and method validation confirm the accuracy of the total and bioaccessible content of dandelion and hibiscus powder capsules.

The in vitro bioaccessible fraction (FBio) of the evaluated elements ranged from 0.13% to 14.31%.

## Introduction

In recent years, the search for alternative food supplements has expanded due to changing consumer behavior, increased life expectancy, and, most importantly, the need for worldwide regulation of these products. Various chemical, pharmaceutical, cosmetic, and food-related substances have been investigated, and unconventional food plants (UFPs) stand out as sustainable alternatives for dietary diversification and therapeutic purposes. In this context, unconventional edible flowers (UEFs) are noteworthy due to their low cost, wide accessibility, promotion of local biodiversity and culture, and contribution to food security [13, 55, 84, 87].

Notable UEFs include dandelion (*Taraxacum officinale*) and hibiscus (*Hibiscus sabdariffa*), which have been traditionally used as food and in herbal preparations [34, 62, 65, 77]. *T. officinale*, commonly known as dandelion, is a herbaceous plant native to North America, Europe, and Asia. It is considered a common weed in gardens, agricultural fields, pastures, and vacant lots. It grows to about 40 cm in height and has yellow to orange flowers and irregularly shaped leaves. The leaves are used in salads, drinks, and vegetable-based dishes due to their high nutritional value. Dandelion is a vegetable that is rich in fiber, minerals, vitamins, and essential fatty acids [2, 43]. *H. sabdariffa* is native to India, easy to cultivate, and suitable for integration into polyculture systems and food production. The dark red petals are commonly used as the main ingredient in fruit teas. The calyx also provides a variety of health benefits [56, 99]. The plant can be grown in many different types of soil, demonstrating its high adaptability [8]. This species is used as a dietary supplement, a product that provides nutrients, active substances, enzymes, or probiotics to complement the diet and increase its nutritional value. Dietary supplements can be found in various forms, such as tablets, capsules, liquids, and powders [27, 48, 76].

The growing market for UFP-based supplements requires more rigorous regulation and quality control, including labeling and nutritional composition, to ensure safety and efficacy. According to the Brazilian National Health Surveillance Agency (ANVISA), a food supplement is defined as an oral product with distinct pharmaceutical forms that is intended to supplement the diet of a healthy population with nutrients, bioactive substances, enzymes, or probiotics, either isolated or combined [22]. Regarding the pharmaceutical forms of food supplements, the market allows for various possibilities. They can be found in solid, semi-solid, or liquid forms, including capsules, tablets, liquids, powders, bars, gels, lozenges, chewing gum, and other forms [25]. In Brazil, dandelion and hibiscus powders are widely sold in capsule form, both online and in stores.

Several studies have demonstrated the nutritional value of UEFs, indicating that they are a good source of digestible proteins, carbohydrates, fiber, microelements, and vitamins, while having a low lipid content [30, 31, 47, 53, 61, 68, 79]. Inorganic constituents comprise between 5 and 10% of a flower's dry biomass and play essential structural and physiological roles in successful flowering and pollination. Microelements, such as iron (Fe), zinc (Zn), and manganese (Mn), act as cofactors in enzymatic systems that protect flowers from oxidative stress and aid in the synthesis of pigments that attract pollinators. UEFs are recognized as a promising source of essential elements, including Fe, Mn, and Zn. When absorbed, these elements contribute to the nutritional and functional value of the human diet. Therefore, proper origin and handling are fundamental to balancing the benefits of minerals and their potential adverse health effects at elevated concentrations [28].

Copper is an essential micronutrient involved in several physiological processes, including oxidative metabolism, connective tissue formation, and antioxidant defense. Copper acts as a cofactor for enzymes such as cytochrome c oxidase and superoxide dismutase, which are involved in mitochondrial respiration and protection against oxidative stress. Adequate copper intake has been associated with proper immune function and cardiovascular health. However, excessive exposure to copper can lead to oxidative damage and hepatic toxicity due to the accumulation of free copper ions in tissues. This underscores the importance of maintaining copper intake within the recommended range [7, 36, 82]. Iron is one of the most important trace elements in human nutrition because it participates in hemoglobin formation, oxygen transport, and various metabolic processes. Iron deficiency is one of the most common nutritional deficiencies worldwide and is associated with anemia, fatigue, and impaired cognitive and immune function [60]. However, excessive iron intake can also be harmful because free iron can catalyze reactions that form free radicals, contributing to inflammatory processes and oxidative damage [81].

Manganese is an essential cofactor for several enzymes involved in energy metabolism, bone formation, and antioxidant defense, including manganese superoxide dismutase. However, high manganese intake, though essential, can cause neurotoxic effects and interfere with the metabolism of other elements, particularly iron. This can alter its absorption and homeostasis in the body. Therefore, consuming foods rich in manganese can pose a risk, especially for women, who have higher iron requirements [98]. Zinc also plays a critical role in human physiology by being involved in the regulation of over 300 enzymatic reactions. It contributes to DNA synthesis, protein metabolism, immune response, and wound healing. Zinc deficiency has been associated with growth retardation, impaired immune function, and increased susceptibility to infections [51]. Conversely, chronic excessive zinc intake can interfere with copper metabolism and disrupt the homeostasis of other essential trace elements, underscoring the importance of balanced intake [85].

In vitro bioaccessibility refers to the fraction of a nutrient or bioactive compound released from its food matrix in systems that simulate the gastrointestinal tract. This process makes the analyte soluble and available for absorption in permeation models that mimic the small intestine [52]. Recent studies use simulated digestion models that replicate the oral, gastric, and intestinal phases by controlling variables such as pH, enzymes (pepsin and pancreatin), and bile salts. These models are used to estimate the utilization of minerals and other chemical species in foods, such as UFPs [32, 39, 41, 73, 90]. The INFOGEST in vitro static simulation protocol of gastrointestinal digestion is currently being used to evaluate the release of inorganic constituents in food matrices [9, 41, 90]. This digestion model is based on available physiological data and describes situations that are as close as possible to the in vivo method [29, 71]. This methodology is considered an efficient, fast, and low-cost predictive tool for nutritional screening. However, despite its broad applicability, this method does not fully represent all processes occurring in vivo, particularly cellular activity, hormonal and nervous control, and immune system involvement [41, 90, 97].

Methods are needed to decompose samples and perform analyses to determine the presence of essential and non-essential elements in the in vitro bioaccessible fraction of food matrices. These methods include conductive heating in a closed digestion block or digestion in an oven with microwave-assisted heating [41, 63, 90]. For biological samples, analytical techniques based on inductively coupled plasma (ICP) are commonly used [45, 83, 84]. Among modern techniques, microwave-induced plasma optical emission spectrometry (MIP OES) has gained interest in recent years due to its diverse applications and suitable analytical performance. MIP OES is a useful tool because its instrumentation and operating costs are low and comparable to those of traditional inductively coupled plasma optical emission spectrometry (ICP OES) and flame atomic absorption spectrophotometry (F AAS), enabling rapid multi-element analysis [50]. This analytical technique is used in various fields of chemical analysis due to its high sensitivity, speed, ability to analyze multiple elements, ease of operation, and high precision and accuracy [16, 38]. In food analysis, a significant issue is the relationship between quality as perceived by consumers and the presence of elements that provide health-promoting properties. In this context, it should be noted that the total and bioaccessible content of UEFs is still unknown, particularly for products sold in natural food stores worldwide [64].

The aim of this study was to evaluate the chemical composition of edible flower capsules containing dandelion and hibiscus using procedures to determine moisture, ash, lipids, and proteins. Additionally, microelements (Cr, Cu, Fe, Mn, Ni, and Zn) were determined simultaneously by MIP OES after digestion in a closed-block system. In vitro bioaccessibility was evaluated using the INFOGEST 2.0 in vitro protocol. The analytical performance of the procedures was statistically evaluated in terms of limits of detection and quantification, precision, and accuracy. No literature was found reporting data on the bioaccessibility of the mineral profile of UEFs – dandelion (*T. officinale*) and hibiscus (*H. sabdariffa*). To the best of our knowledge, this is the first study to specifically evaluate the bioaccessible trace element content of these edible flowers using the INFOGEST 2.0 in vitro protocol and MIP OES to assess microelements.

## Materials and Methods

### Sampling and Labeling Analysis

Samples of UEF-based supplements for two species (*T. officinale* and *H. sabdariffa*) were commercially acquired from three different manufacturers in Brazil through access and purchases in the digital market. The supplements were solid oral hard capsules containing 500 mg and did not contain other substances (excipients). For identification purposes, the samples were coded as follows: DFV, DNN, DPN, HFV, HNN, and HPN. The capsule contents were homogenized using a mortar and pestle. All samples were preserved and stored in plastic containers in desiccators until analysis.

The products' labels were evaluated in accordance with the provisions set forth in Resolution No. 243/2018 and Instruction No. 28/2018 of the Collegiate Board (RDC), both of which are from the Brazilian Ministry of Health. These documents establish lists of constituents, limits of use, sanitary requirements, and additional supplement claims [22, 23]. Additionally, RDC No. 727/2022, which addresses the labeling of packaged foods, was consulted [25].

### Instrumentation and Operational Parameters

The moisture content was measured gravimetrically for the centesimal composition, considering all volatile components at 105 °C in an oven until a constant weight was reached (Quimis® Q317 M33, Diadema, São Paulo, Brazil). The fixed mineral residue was obtained by calcining approximately 3 g of the sample at 550 °C for about six hours in a muffle furnace (Quimis® Q318 M24, Diadema, São Paulo, Brazil). Lipid extraction was based on a continuous process using a Soxhlet extractor (Solab®, SL-145 V, Piracicaba, Brazil) for eight hours on a heating plate at 80 °C with petroleum ether. This was followed by solvent evaporation. The protein content was determined using the Kjeldahl method with a digestion block (Marconi®, MA-4025, Piracicaba, São Paulo, Brazil). Nitrogen distillation was performed using a Marconi nitrogen distiller (Marconi®, M-036, Piracicaba, São Paulo, Brazil) and excess boric acid (4%) to produce ammonia. Total carbohydrate content was calculated by subtracting the sum of the percentages of moisture, ash, lipids, and proteins from 100. The moisture, ash, protein, lipid, and carbohydrate values were determined according to the standards methods of the Association of Official Analytical Chemists [3]. All chemical analyses were performed in triplicate.

The samples were weighed to an accuracy of 0.001 g (± 0.0001) using an analytical balance (Shimadzu®, model ATX224R, Kyoto, Japan). A closed digestion block (Tecnal®, model 015–1, São Paulo, Brazil) equipped with 15 polytetrafluoroethylene tubes (TMF®, São Paulo, Brazil), which were modified for digestion and coupled to a temperature controller (Tecnal®, model E-007 MOP, São Paulo, Brazil), was used for the total acid digestion of the samples. A centrifuge (FANEM^®^, model 2206, São Paulo, Brazil) and a pH meter (Gehaka^®^, model PG2000, São Paulo, Brazil) were used for separation and pH adjustment, respectively. Analytical determinations were performed using a Microwave Induced Plasma Optical Emission Spectrometer (MIP OES) spectrometer (Agilent Technologies^®^, model 4210, Santa Clara, CA, USA). The nebulizer features a double-pass glass cyclonic spray chamber system and a standard torch with an axial viewing position. A nitrogen generator (Agilent Technologies^®^, Model 4107, Santa Clara, CA, USA) was used to remove ambient air via a Rotor Plus air compressor (Metalplan Airpower^®^, São Paulo, Brazil). The instrumental parameters were as follows: peristaltic pump rotation at 25 rpm; stabilization time at 15 s; capture time at 35 s, with three repetitions; and a variable nebulization flow rate (L min⁻^1^) for each element: Cr (0.90), Cu (0.70), Fe (0.65), Mn (0.90), Ni (0.70), and Zn (0.45). The selected spectral lines (nm) corresponded to those with the highest intensity for each analyte: Cr (425.433), Cu (324.754), Fe (371.993), Mn (403.076), Ni (352.450), and Zn (213.857).

### Reagents, Solutions and Decontamination of Materials

All reagents were of analytical grade, and solutions were prepared with ultrapure water (specific resistivity of 18 MΩ·cm⁻^1^) from a Milli-Q^®^ purification system (Millipore^®^, Bedford, MA). The reagents used for acid digestion of the samples included 65% (m m^−1^) Suprapure HNO₃ (Merck^®^, Darmstadt, Germany), 30% (m/m) H₂O₂ (Vetec^s^, São Paulo, Brazil), and ultrapure water. Multielement solutions were prepared from 1000 mg L^−1^ SpecSol^®^ monoelement solutions of Cr, Cu, Fe, Mn, Ni, and Zn. All reagents and enzymes used in the INFOGEST 2.0 in vitro protocol [29] were purchased from Merck®, Darmstadt, Germany.

A multielement stock solution (50 mg L⁻^1^) containing the microelements (Cr, Cu, Fe, Mn, Ni, and Zn) was prepared to construct the analytical curve. This solution was made from a single-element stock solution (1000 mg L^−1^). To obtain 100 mL of the 50 mg L^−1^ stock solution, 5 mL of each single-element standard solution was transferred to a 100-mL volumetric flask and the volume was made up with 5% (v/v) nitric acid. The calibration curve was prepared by appropriately diluting the stock solution, yielding 12 points with concentrations ranging from 0 to 7 mg L^−1^. To ensure all measurements remained within the linear calibration range, the original digested samples were diluted according to specific dilution factors (DF) prior to MIP OES analysis. These factors varied according to the elemental concentration of the supplements, ranging from 1.7- to 250-fold.

The glassware and materials were decontaminated in a 10% (v v^−1^) HNO₃ solution in a water bath for 24 h. The TMF® tubes used for digestion were decontaminated in a closed digestion block at 180 °C for one hour and fifteen minutes using 10 mL of 65% (m m^−1^) nitric acid. After decontamination, all glassware, tubes, and materials were thoroughly washed with ultrapure water.

### Digestion Procedure and Total Content of Inorganic Constituents in UEF Samples

The samples were digested using a method previously validated by our research group [38]. Approximately 0.5 g of each sample was added to a reaction flask in the closed-system digestion block. Then, 2 mL of 65% (m m^−1^) HNO₃ and 2 mL of 30% (m m^−1^) H₂O₂ were added, followed by 4 mL of ultrapure water. The samples were heated to 200 °C for 75 min. Then, the flasks were cooled to 25 °C. The digested samples were transferred to volumetric flasks, and the final volume was adjusted to 25.0 mL with deionized water. The microelement content of Cr, Cu, Fe, Mn, Ni, and Zn was determined by MIP OES. All analyses were performed in triplicate. Blank analytical solutions were prepared using the same procedure.

### Bioaccessibility of Inorganic Constituents in UEF Samples (INFOGEST 2.0 in Vitro Protocol)

Microelement bioavailability estimates (Cr, Cu, Fe, Mn, Ni, and Zn) were performed based on the INFOGEST 2.0 in vitro protocol [41, 90]. The protocol involves solubilizing the sample in simulated salivary fluid containing (KCl, KH₂PO₄, NaHCO₃, MgCl₂(H₂O)₆, (NH₄)₂CO₃, HCl, and CaCl₂), followed by exposure to salivary amylase for two minutes. Then, simulated gastric fluid containing gastric enzymes (pepsin and lipase) was added. In addition to gastric enzymes, this fluid also contained KCl, KH₂PO₄, NaHCO₃, MgCl₂(H₂O)₆, (NH₄)₂CO₃, NaCl, HCl, and CaCl₂. The mixture was then incubated at pH = 3.0 for two hours at 37 °C. Finally, the mixture was exposed to simulated intestinal fluid containing pancreatin and bile [KCl, KH₂PO₄, NaHCO₃, MgCl₂(H₂O)₆, NaCl, HCl, and CaCl₂] at pH = 7.0 and 37 °C for two hours. After these steps, the samples were centrifuged at 3,000 rpm for four minutes. The resulting supernatant was filtered through a 0.45 µm PTFE syringe filter and analyzed by MIP OES. All analyses were performed in triplicate.

Due to the limited sample size, the in vitro bioaccessible fraction (BF) was only determined in two UEF samples: DFV and HNN. The BF was calculated using an equation adapted from that proposed by Tokalioglu et al. [96]:

$$\mathrm{\%}\mathrm{B}\mathrm{F} = (\mathrm{F}\mathrm{B}\mathrm{C} / \mathrm{T}\mathrm{T}) \times 100\mathrm{\%}$$

where: %BF is the percentage of bioaccessible fraction; FBC is the fraction of inorganic constituents in the supernatant; and TT is the total content of inorganic constituents (determined individually).

The solid residues from the capsules containing dandelion (*T. officinale*) and hibiscus (*H. sabdariffa*) were digested in a closed digestion block using the same procedure as total digestion. The digests were adjusted to 25 mL with ultrapure water, and the bioaccessible fractions of Cr, Cu, Fe, Mn, Ni, and Zn were determined by MIP OES.

The parameters of linearity, matrix effect, accuracy, precision, and limits of detection (LOD) and quantification (LOQ) were evaluated [94]. The linear equations and coefficients of determination indicated good method linearity (R^2^ ≥ 0.998). The matrix effect was evaluated using the ratio of the slopes of the linearity and spiked sample curves, which covered the same concentration ranges [21]. Accuracy was assessed via addition and recovery tests at three concentration levels (0.5,3.0, and 6.0 mg L^−1^). Precision was verified by the repeatability of the results observed within and between days and was expressed as the relative standard deviation (% RSD). When calculating the LOD and LOQ, the background equivalent concentration (BEC) and signal-to-background ratio (SBR) were considered:

$$\begin{array}{c}\mathrm{S}\mathrm{B}\mathrm{R}\;=\;({\mathrm{I}}_\mathrm{P}\;-\;{\mathrm{I}}_\mathrm{B})\;/{\mathrm{I}}_\mathrm{B}\\\mathrm{B}\mathrm{E}\mathrm{C}\;=\;{\mathrm{C}\mathrm{I}}_\mathrm{P}/\mathrm{S}\mathrm{B}\mathrm{R}\\\begin{array}{c}\mathrm{L}\mathrm{O}\mathrm{D}\;=\;(3\;\mathrm{x}\;{\mathrm{R}\mathrm{S}\mathrm{D}}_\mathrm{B}\;\mathrm{x}\;\mathrm{B}\mathrm{E}\mathrm{C})\;/\;100\\\mathrm{L}\mathrm{O}\mathrm{Q}\;=\;(10\;\mathrm{x}\;{\mathrm{RSD}}_{\mathrm B}\;\mathrm{x}\;\mathrm{B}\mathrm{E}\mathrm{C})\;/\;100\end{array}\end{array}$$

where, I_P_ is the emission intensity at a given point on the calibration curve; I_B_ is the average emission intensity of the analytical blanks (i.e., the reagents used in the absence of the matrix after method optimization), and CI_P_ is the concentration corresponding to the emission intensity at the chosen point on the calibration curve. Additionally, the INFOGEST 2.0 in vitro protocol was validated via the mass balance of the elemental concentrations determined in the bioaccessible fraction and residue. The sum of these fractions was then compared to the total concentration of each element in the samples. All analyses were performed in triplicate. Blank analytical solutions were prepared using the same procedure.

### Statistical Analysis

Statistica 7.1 software (Statsoft®, Inc.) was used to evaluate the multielement and proximate composition data using a completely randomized design. The data were analyzed at a 95% confidence level, and results were considered statistically significant when p ≤ 0.05. The statistical test was selected based on the number of quantifiable groups being compared within the same species. For comparisons involving three groups, a one-way analysis of variance (ANOVA) was performed, followed by a Tukey post hoc test to identify differences between means. When the analyte concentration in a sample was below the limit of detection (LOD), leaving only two groups to be compared, a Student's t-test was used.

## Results e Discussion

### Sanitary Registration and Labeling Analyses

None of the samples used in the research were registered with the Brazilian Ministry of Health. The companies claim on their product packaging that they are exempt from registration under certain Brazilian laws [17, 18, 20, 22, 24]). This study analyzed supplement labels to determine compliance with Brazilian legislation [22, 23, 25]. All analyzed products (100%) met the following requirements: 1) the name of the source from which the nutrient, bioactive substance, or enzyme was extracted, 2) the recommended use, 3) the warning "Keep out of reach of children" written in bold, prominent text, 4) the storage instructions, including those for after opening the package, and 5) the serving size as declared in the nutritional information. However, none of the products met the requirements for designation as a "food supplement." This designation must be declared near the product brand in legible characters that meet the declaration requirements. Approximately 67% of the products did not meet the requirements: 1) A prominent, bold warning stating "This product is not a medicine"; and 2) A prominent, bold warning stating "Do not exceed the recommended daily intake indicated on the packaging." Additionally, 33% did not meet the requirement for textual variations of authorized claims.

The labeling assessment revealed significant nonconformities in all analyzed products.

According to RDC No. 243/2018 [22], all six products failed to comply with Articles 12 and 13 because they did not display the phrase "Food Supplement" in uppercase and bold letters. Additionally, two samples lacked the warnings required by Article 14: "This product is not a medicine," and "Do not exceed the recommended daily intake indicated on the packaging". These errors compromise both the clarity of communication and consumer safety. Errors in the labeling of food supplements are common and include the absence of mandatory warnings and incorrect designations. Some labels even mention therapeutic properties. Arévalo and Sánchez [4] analyzed 130 supplement labels sold online and found that 61.5% did not display the designation "food supplement" next to the pharmaceutical form. Furthermore, 76.2% of the supplements failed to include mandatory warnings in bold, such as "This product is not a medicine" and "Do not exceed the recommended daily intake indicated on the packaging".

Regarding RDC No. 727/2021 [25], two samples made claims about having medicinal, therapeutic, or disease-preventive properties. However, this is in disagreement with Article 4, which expressly prohibits this type of information on food products. This irregularity is considered serious because it may mislead consumers into believing that the supplements are pharmacologically effective, exceeding the legally recognized purpose of this product category [72]. These nonconformities reflect failures to comply with legislation and pose potential risks to public health, as labeling is one of the main instruments for informing consumers. Inadequate labeling compromises the transparency, traceability, and credibility of food supplements, constituting a sanitary infraction subject to legal penalties [26, 72]. Therefore, these results underscore the necessity of intensified oversight and greater accountability from companies in complying with current regulations.

With the wide variety of food supplements available, it is evident that properly evaluating labels and verifying their compliance with quality standards, sanitary requirements, definitions, composition rules, and safety and quality regulations is important [44, 86]. Nutritional labeling informs consumers about the quality of these products. The Brazilian Ministry of Health, through ANVISA, has unified the categories of vitamin and mineral supplements, bioactive substances, probiotics, novel foods, foods with functional property claims, supplements for athletes, and food supplements for pregnant and breastfeeding women, and specific over-the-counter medications. This unification has enabled the government to accomplish goals such as ensuring the safe, accessible, and high-quality consumption of food supplements, reducing information asymmetry in the market, facilitating sanitary control and risk assessment of products, eliminating unnecessary obstacles to commercialization and innovation, and streamlining the existing regulatory framework [26].

### Proximate Composition

The proximate composition of dandelion and hibiscus capsules varied among different suppliers (Table 1). These variations may be due to differences in the drying process, the plants' origin, or storage conditions [95]. Statistically significant differences in moisture, ash, protein, and carbohydrate content were observed among the samples (*p* < 0.05). The moisture content of all samples was within the maximum limit of 15% established by RDC No. 263/2005 [19]. This is a relevant parameter since moisture directly affects product stability, quality, and chemical composition, as well as favoring microbiological deterioration. In powdered samples, such as powders and flours, moisture content can affect particle size distribution and apparent density [80, 91].

**Table 1:** The centesimal composition (in, %) of the capsules contains dandelion (*T. officinale*) and hibiscus (*H. sabdariffa*) powder

Parameter/Samples	DFV	DNN	DPN	HFV	HNN	HPN
Moisture	6.52 ± 0.01^b^	8.61 ± 0.06^c^	5.68 ± 0.01^a^	5.73 ± 0.01^a^	12.00 ± 0.20^c^	8.04 ± 0.01^b^
Ash	4.35 ± 0.05^b^	3.48 ± 0.04^a^	6.08 ± 0.07^c^	15.70 ± 0.04^c^	12.62 ± 0.03^b^	4.70 ± 0.06^a^
Protein	0.44 ± 0.00^b^	0.35 ± 0.03^a^	0.81 ± 0.01^c^	0.64 ± 0.01^a^	0.62 ± 0.01^a^	0.70 ± 0.01^b^
Lipids	1.72 ± 0.10^b^	1.89 ± 0.01^b^	1.12 ± 0.08^a^	1.83 ± 0.08^a^	2.34 ± 0.51^a^	1.85 ± 0.44^a^
Carbohydrates	87.00 ± 0.14^c^	86.31 ± 0.14^b^	86.71 ± 0.16^b^	76.12 ± 0.12^b^	72.42 ± 0.27^a^	84.8 ± 0.4^c^

The values are presented as the average of triplicates ± standard deviation (SD). A statistical test was performed to compare samples of the same species. According to Tukey's test at a 95% confidence level, means with the same lowercase letter do not differ statistically

The literature contains more studies on the use of the plant in its natural (fresh state), as well as on the use of its various parts, such as the leaves, roots, and flowers. In this study, the analyzed contents were dry and inside the capsules. No literature was found with these values for the powder content of the UEFs capsules studied. It should be noted that the encapsulated material underwent several processes, including drying, which can alter the composition.

The dandelion samples had a moisture content ranging from 5.68% to 8.61%. The HNN sample had the highest moisture content (12.00%). Changes in the fresh weight of flower stems after harvest have been documented in the literature [57]. Almeida et al. [2] and Biel et al. [14] reported moisture contents of approximately 88.5% and 89.4%, respectively, in fresh leaves from Minas Gerais (Brazil), and Lipnik (Poland). Colussi et al. [37] and Khan et al. [59] observed higher ash content (15.53% and 11.28%, respectively) in dandelion flour and leaves from Rio Grande do Sul (Brazil) and Khyber Pakhtunkhwa (Pakistan) than in the samples examined in this study. Biel et al. [14] and Almeida et al. [2] noted considerable diversity in ash content in dandelion leaves (4.2%−14.8% and 1.43%, respectively).

The protein levels in the dandelion samples were significantly lower (0.44%−0.81%) than the 15.96% reported by Colussi et al. [37], who used the same Kjeldahl method with a conversion factor of 6.25. Similarly, Murtaza et al. [75] reported 16.01% in shade-dried samples from Koshmir (India). There were no significant differences in lipid content between DFV and DPN (1.72% and 1.89%, respectively). However, the lipid content of these samples differed statistically from that of DPN (1.12%). The lipid content of the studied dandelion samples was lower than the values reported by Colussi et al. [37] (3.66%) and Murtaza et al. [75] (4.49%). The results were similar to those of Almeida et al. [2], who reported a lipid content of 1.20 ± 0.03% in fresh dandelion leaves. The analysis of carbohydrate content was performed on dry samples using the difference method, resulting in high values (84.8%), which contribute to the caloric value of the analyzed samples.

No statistically significant difference (*p* ≥ 0.05) was observed between the HFV (1.83%) and HPN (1.85%) hibiscus samples regarding lipid content. However, these samples differed statistically from the HNN sample (2.34%). Additionally, no significant difference in protein content was found between the HFV (0.64%) and HNN (0.62%) samples. Statistically significant differences (p ≤ 0.05) were observed in the moisture, ash, and carbohydrate content. Particular emphasis was placed on the high ash content observed in the HFV (15.70%) and HNN (12.62%) samples. Ash, the inorganic fraction of food composed of minerals essential to cellular metabolism, also showed significant variation. Abreu et al. [1] reported an ash content of 8.6% in dried hibiscus from Piauí (Brazil), which is lower than the values found in two samples (HFV and HNN) in the present study, but higher than the value obtained in HPN (4.7%). These results reinforce the idea that the drying process can concentrate minerals in the plant matrix, thereby increasing their content compared to fresh species. There was no significant difference in protein levels between HFV and HNN. However, the protein content in these samples was lower than in HPN. These results were higher than the 0.2% reported by Abreu et al. [1] in vegetable flour. Lipid levels were higher than those reported by Abreu et al. [1] and Botrel et al. [15] in hibiscus samples from Piauí and the Federal District of Brazil, at 0.85% and 0.65%, respectively. Keyata et al. [58] evaluated the proximal composition of reddish-brown calyces and dried seeds of Karkade from Ethiopia's Benishangul-Gumaz region, reporting the following percentages: ash (5.2%−13.1%), protein (4.55%−28%), lipids (1%−18%), and carbohydrates (32.8%−67.7%). Our findings are consistent with those of the aforementioned authors. Additionally, the results of the present study are consistent with those of Edo et al. [46], who reported the proximate composition of dried hibiscus leaves and calyces (moisture content: 12.5%,ash content: 14.5%) in a review article.

### Multielement Analysis in Capsule Samples Containing Dandelion and Hibiscus

The chemical elements identified in the samples play crucial roles in plant development and human homeostasis. In plants, these micronutrients are involved in essential enzymatic and structural processes. In humans, they are essential for oxygen transport, modulation of the immune response, and various metabolic and antioxidant reactions [42]. Consequently, even trace amounts of these elements enhance the nutritional value of flowers, underscoring the potential of UEFs as a dietary source of microelements [6, 70].

As shown in Table 2, the multielement analysis of dandelion and hibiscus capsule contents revealed variations among different suppliers. No literature was found on multielemental analyses of edible flowers using MIP OES. Therefore, our work is pioneering and makes a relevant scientific contribution. Overall, comparing our results with those of previous studies highlights both consistencies and divergences in the elemental composition of UEFs. This high variability may be associated with the harvest period and other environmental conditions affecting the chemical composition of the cultivation site [92, 100].

**Table 2:** Multi-elemental composition of capsules containing Dandelion (*T. officinale*) and Hibiscus (*H. sabdariffa*)

Sample	Elements (µg g^−1^)					
	Cr	Cu	Fe	Mn	Ni	Zn
DFV	0.62^a^ ± 0.01	1.20^A^ ± 0.13	159.44^a^ ± 1.50	441.34^c^ ± 6.00	1.29^a^ ± 0.07	30.98^B^ ± 1.20
DNN	0.70^a^ ± 0.06	< 0.13*	182.00^a^ ± 16.00	48.94^a^ ± 3.00	1.11^a^ ± 0.12	< 0.54*
DPN	0.85^b^ ± 0.04	11.45^B^ ± 0.87	567.00^b^ ± 11.00	102.50^b^ ± 1.70	1.66^b^ ± 0.02	1.60^A^ ± 0.07
HFV	2.82^B^ ± 0.12	6.51^c^ ± 0.11	1880.90^c^ ± 152.00	139.50^b^ ± 3.20	< 0.24*	17.40^b^ ± 1.30
HNN	1.92^A^ ± 0.02	4.70^b^ ± 0.07	1033.00^b^ ± 13.00	376.30^c^ ± 2.00	3.21^B^ ± 0.03	23.50^c^ ± 1.90
HPN	< 0.44*	3.14ª ± 0.07	190.00^a^ ± 4.00	89.00^a^ ± 1.00	1.96^A^ ± 0.03	11.25^a^ ± 0.48

Means with the same lowercase letters in the same column do not differ statistically by Tukey's test at the 95% confidence level (*p* ≥ 0.05)

Means with the same uppercase letters in the same column do not differ statistically by Student's t-test at the 95% confidence level (*p* ≥ 0.05)

The statistical test comparison was performed between samples of the same species

*: Below of the quantification limit (LOQ)

Among the dandelion samples analyzed, no statistically significant differences (*p* ≥ 0.05) were observed in the levels of Cr or Ni between DFV and DNN. Specifically, no significant differences were observed in the levels (in µg g^−1^) of Cr between DFV (0.62) and DNN (0.70), nor in the levels (in µg g^−1^) of Ni between DFV (1.29) and DNN (1.11). Studies confirmed the presence of these microelements in *T. officinale* in quantities equivalent to those observed in this study. Soares and Quináia [89] used F AAS to determine the microelement levels (in µg/100 g) of Fe (170–840), Mn (130–890), and Zn (40–80) in dried dandelion flowers from Paraná (Brazil). The results of the present study align with those of [33], except for Zn, for which the values were lower. Almeida et al. [2] determined the concentrations (in mg kg^−1^) of microelements in fresh leaves collected in Minas Gerais (Brazil), using ICP OES. The results showed the following concentrations: Cu (0.08 ± 0.01), Fe (2.35 ± 0.27), Mn (0.25 ± 0.00), and Zn (0.6 ± 0.02). In the current study, we found higher concentrations of Fe and Mn. Biel et al. [14] reported the following concentrations (in mg kg^−1^) of microelements in fresh dandelion leaves from Lipnik (Poland): Cu (0.39–0.55), Fe (4.60–14.10), and Zn (3.76–3.99). The Cu and Zn content is consistent with the concentration range found in capsule samples containing dandelion.

Medeiros et al. [69] used F AAS to determine Cu, Mn, and Zn during the optimization of extraction conditions, and used ICP OES for multielement analyses. For dandelion leaves (*T. officinale*) collected in Minas Gerais (Brazil), the following concentrations were found (mg kg^−1^): Cu (0.13), Mn (21), and Zn (77). The present study found higher levels of Mn and Zn in the studied capsules than the previous study did. Additionally, Silva et al. [88] analyzed microelements in dried material from various unconventional edible plants sold in specialty pharmacies and drugstores in São Paulo (Brazil). The authors used instrumental neutron activation analysis (INAA) and ICP OES to determine Cr, Fe, Mn, and Zn, and used cold vapor atomic absorption spectrometry (CV AAS) to quantify Cu and Ni. The contents (in mg kg^−1^) were: Cr (9.6 ± 0.7), Cu (7.04 ± 0.06), Fe (1,979 ± 25), Mn (128 ± 4), Ni (2.6 ± 0.2), and Zn (63 ± 6). In the present study, the contents of Cr, Fe, Ni, and Zn were lower, while Cu and Mn remained consistent. Khan et al. [59] determined the following concentrations of elements in dandelion from Khyber Pakhtunkhwa (Pakistan) (in mg/100 g): Cu (0.05 ± 0.05), Fe (1.671 ± 0.133), and Zn (0.141 ± 0.04). The Cu and Zn content was consistent with that of the aforementioned authors, while the Fe content was lower. Changes in the mineral content of a species may be influenced by geographic variations related to soil type, climate, growing conditions, and the storage and transport conditions of the samples [92, 100].

For the hibiscus samples, the determined values showed significant variation among different manufacturers (p ≤ 0.05). Malik et al. [67] found the following concentrations (µg g^−1^) by ICP OES: Fe (219.8), Mn (672.5), Ni (7.92), and Zn (58.39). Most elements had lower concentrations than those obtained in this study, except for Fe, which was higher for the HFV and HNN samples.

Our findings are consistent with those in the literature. Bassey et al. [10] used F AAS to evaluate the content (in mg/100 mL) of Cu (0.68–2.57), Fe (0.34–1.30), Mn (0.15–0.55), and Zn (0.69–1.59) in juices prepared from mature, dried *H. sabdariffa* petals obtained from a local market in Calabar, Cross River State, Nigeria. Bayang et al. [11] determined (in mg kg^−1^) the Fe content (65.5 ± 0.03) on a fresh weight basis using F AAS. Keyata et al. [58] found (in mg kg^−1^) Fe (44–145) and Zn (10–32) content in the calyces and seeds of hibiscus from the Benishangul-Gumuz region of Ethiopia using F AAS. They carbonized the samples on a heating plate, dried them in a muffle furnace, and dissolved them in 5 mL of 6 mol L^−1^ HCl. Similar to our study, Benutić et al. [12] evaluated the concentrations (in mg kg^−1^) of Cr (598 ± 106), Fe (8.61 ± 0.03), Mn (454 ± 11), Ni (3.90 ± 0.09), Cu (2.32 ± 0.12), and Zn (8.19 ± 0.09) in a powdered herbal dietary supplement from the USA (country of manufacture) containing extracts of *Camellia sinensis*, *Malva sylvestris*, *Hibiscus sabdariffa* L., *Elettaria cardamomum*, and lemon extract, which was found on the Zagreb (Croatian) market. They performed the evaluation using inductively coupled plasma mass spectrometry (ICP-MS) after acid digestion in a microwave oven with 5.0 mL of concentrated HNO₃ (69%, w v^−1^), 2.0 mL of distilled deionized water, and 2.0 mL of H₂O₂ (30%, v v^−1^).

In Brazil, the Brazilian Food Composition Table [93] compiles data on UFPs, including edible flowers from local, regional, and national sources. Our research group contributed to this repository [74]. The data obtained in this study can serve as a basis for updating the TBCA and expanding knowledge about alternative sources of microelements. Dandelion and hibiscus are widely distributed plants gaining prominence as potential food supplements in commercially available capsule products. However, despite sharing the same matrix, levels of these microelements can vary significantly among manufacturers due to factors such as production processes, drying of raw materials, and contamination during processing.

### In vitro Bioaccessibility of Inorganic Constituents in UEFs Samples

There is a lack of studies on the in vitro bioaccessibility of nutrients, such as bioactive compounds and inorganic constituents, in UEFs and other non-conventional foods in the literature [39–41, 41, 49, 78, 90, 90]. The bioaccessibility of microelements depends on their solubility and release from the food matrix during digestion [35]. No studies employing the INFOGEST method for the in vitro digestion of UEFs or related to bioaccessible mineral fractions using MIP OES or other spectroanalytical techniques were found in the literature. Therefore, this study is pioneering in the field of bioaccessibility research with these food matrices, particularly dandelion and hibiscus.

No significant variations were observed in the analytical signals of the measurements between the analytical curves using an acidic medium and those from the bioaccessibility assays. The same was true for samples derived from total acid digestion and bioaccessibility procedures. The matrix effect study showed a ratio of up to 1.0 between the curves, indicating an absence of matrix interference in the analyses. The limits of detection (LOD) and quantification (LOQ), expressed in mg kg^−1^, refer to the determination of the in vitro bioaccessible fraction of inorganic constituents by MIP OES in UEFs samples (Table 3). Analytical calibration curves for Cr, Cu, Fe, Mn, Ni, and Zn (R^2^ ≥ 0.998) were obtained by diluting appropriate volumes of a multielement standard solution in the analytical blank used for the bioaccessibility test. The measurement precision was less than 10%, indicating good repeatability for microelement analysis in UEFs. The average bioaccessible inorganic constituent content and recovery percentages were obtained from three analytical replicates of the dandelion and hibiscus samples, as shown in Table 4.

**Table 3:** Detection and quantification limits (in mg kg^−1^) for the analytical blank of the in vitro bioaccessibility (INFOGEST 2.0 protocol) and the accuracy tests in UEFs samples

Analyte	LOD (mg kg^−1^)	LOQ (mg kg^−1^)	Accuracy % (addition and recovery)		
			Levels (μg L^−1^)		
			0.5	3.0	6.0
Cr	0.13	0.44	115 ± 4	107 ± 3	110 ± 4
Cu	0.04	0.13	99 ± 3	96 ± 2	97 ± 2
Fe	0.08	0.27	113 ± 6	103 ± 3	107 ± 3
Mn	0.04	0.13	109 ± 2	110 ± 3	111 ± 5
Ni	0.07	0.24	107 ± 4	97 ± 3	99 ± 2
Zn	0.16	0.54	106 ± 3	94 ± 1	91 ± 2

**Table 4:** Concentration of in vitro bioaccessible fractions, residues, and total contents (in mg kg^−1^), bioaccessible fraction, and recovery (in %), determined by mass balance for UEFs samples

**Dandelion capsules poder (DFV sample)**					
**Analyte**	**TT (mg kg**^**−1**^**)**	**FBio (mg kg**^**−1**^**)**	**FBio (%)**	**Residues (mg kg**^**−1**^**)**	**Recovery (%)**
Cr	0.62 ± 0.01	< 0.44	-	0.61 ± 0.14	98.4
Cu	1.20 ± 0.13	0.05 ± 0.02	4.20	0.95 ± 0.09	83.3
Fe	159.4 ± 1.5	< 0.27	-	171.2 ± 10.7	107.4
Mn	441 ± 6	50.6 ± 1.2	11.50	396.5 ± 0.6	101.3
Ni	1.29 ± 0.07	< 0.24	-	1.27 ± 0.19	98.5
Zn	31.0 ± 1.2	1.58 ± 0.31	5.10	24.5 ± 1.5	84.2
**Hibiscus capsules poder (HNN sample)**					
**Analyte**	**TT (mg kg**^**−1**^**)**	**FBio (mg kg**^**−1**^**)**	**FBio (%)**	**Residues (mg kg**^**−1**^**)**	**Recovery (%)**
Cr	1.92 ± 0.02	< 0.44	-	1.82 ± 0.01	94.8
Cu	4.70 ± 0.07	1.17 ± 0.01	3.80	3.94 ± 0.11	108.7
Fe	1033 ± 13	1.33 ± 0.18	0.13	976 ± 12	94.6
Mn	376.3 ± 2.0	45.66 ± 0.55	12.13	275.1 ± 2.5	85.2
Ni	3.21 ± 0.03	0.46 ± 0.01	14.31	2.840 ± 0.001	102.8
Zn	23.5 ± 1.9	1.02 ± 0.04	4.40	18.0 ± 0.7	80.8

*FBio* bioaccessible fraction, *TT* total analyte content

The method of quantifying the bioaccessible in vitro fraction of UEFs was also validated via mass balance. This was achieved by comparing the total elemental content to the solubilized fractions following simulated gastrointestinal digestion. In the absence of specific validation guidelines in the INFOGEST 2.0 in vitro protocol, a margin of 80% to 120% was adopted as the criterion for analytical consistency based on the recovery percentage. This margin is widely accepted in the literature because it accounts for variability in digestive simulations and ensures robust agreement between analyzed fractions [63, 96].

For dandelion, only the bioaccessible fractions of Cu (4.20%), Mn (11.50%), and Zn (5.10%) were obtained. In contrast, the bioaccessible fractions obtained from hibiscus capsule powder samples after in vitro digestion ranged from 0.10% to 4.40% for Cu, Fe, and Zn. The elements with the highest bioaccessible percentages were Mn (12.13%) and Ni (14.31%). This indicates a variation between the total content and the potential availability profile for absorption. Factors that can interfere with nutrient release during digestion include antinutritional compounds such as lectins, glucosinolates, phytates, oxalates, tannins, and saponins. These substances can negatively affect in vitro bioaccessibility by forming insoluble complexes [5, 36]. These results underscore the importance of considering the food matrix composition as a modulator of mineral bioaccessibility in UEFs.

Although the results obtained for FBio (%) are relatively low, they suggest that this matrix could be a potential source of these microelements. Ferreira et al. [49] evaluated the total and bioaccessible concentrations of Cu, Fe, Mn, and Zn in mirindiba pulp and peel using F AAS after digestion. The authors reported that none of the elements became bioaccessible via the INFOGEST in vitro protocol, with concentrations below the method's LOD. Machado et al. [66] studied the variation (29.2%−82.3%) of the bioaccessible fractions of Cu, Zn, Ni, and Fe in raw globe artichoke heads collected from various locations in Uruguay using F AAS and electrothermal atomic absorption spectrometry (ET AAAS). Bioaccessibility experiments were carried out using a simulated in vitro gastrointestinal digestion flow system. The order of FBio (%) from highest to lowest is as follows: Cu > Zn > Ni > Fe.

UEFs can be a potential dietary source of nutrients, such as Cu, Fe, Mn and Zn, that support fundamental physiological functions. However, it is necessary to ensure that these elements remain within established safety limits given that high concentrations of trace elements are associated with adverse health effects. Based on the obtained data, the contribution of the bioaccessible fraction of these elements was calculated in relation to the recommended daily intake (RDI) values according to the Summary Report of Dietary Reference Intakes and Normative Instruction No. 75/2020 (Brazil, 2020), which establishes technical requirements for the nutritional labeling of packaged foods. Reports from the Institute of Medicine's Food and Nutrition Board [54] were also referenced. The nutritional composition of the evaluated UEFs is presented in Table 5. To facilitate direct comparison with other ingredients and comply with standard nutritional labeling practices, all elemental concentrations are expressed based on a standardized 100-g portion. According to these results, this level of consumption would moderately contribute to Cu, Fe, and Zn intake, while providing a very high Mn contribution that exceeds daily recommendations in some scenarios. Generally, the intake of these elements through plant-based foods is considered safe and beneficial since these foods provide micronutrients at physiological levels in complex food matrices that modulate their bioavailability. However, evaluating the bioaccessible fraction, as this study does, is essential to estimating the actual nutritional contribution and avoiding interpretations based solely on total mineral concentration. The results show that the quantified levels are within the limits set out in the referenced standards. These results suggest that these UEFs could complement the dietary intake of essential elements. Based on their nutritional profile, these samples could serve as an additional source of micronutrients in a balanced diet. Further testing is required to evaluate in vitro*-*in vivo correlations using permeation and bioavailability study models.

**Table 5:** Average contribution of the bioaccessible fraction* of minerals present in edible flowers to the daily intake recommendations, considering differences between sexes and values established by different official institutions

Element	Men bioaccessible fraction (mg/100 g)	RDI – Men (NIH)	%RDI cont. – Men	RDI – Women (NIH)	%RDI cont.—Women	RDI – ANVISA*	%RDI cont
Cu	0.061	0.9^RDA^	6.8%	0.9^RDA^	6.8%	0.9	6.8%
Fe	0.067	8.0^RDA^	0.8%	18.0^RDA^	0.4%	14.0	0.5%
Mn	4.813	2.3^AI^	209.3%	1.8^AI^	267.4%	2.3	209.3%
Zn	0.130	11.0^RDA^	1.2%	8.0^RDA^	1.6%	7.0	1.9%

*Average contribution of the bioaccessible fraction: corresponds to the amount of each element released after in vitro digestion, considering only species validated by the mass balance approach. RDI: Recommended Daily Intake for healthy adults aged 19 to 50 years; AI: Adequate Intake; ADI: Recommended Daily Allowance. ANVISA: reference values applied for nutritional labeling without differentiation by sex; NIH: reference values established with specific recommendations by sex. %RDI contribution: estimated percentage contribution to the daily intake of minerals based on the consumption of 100 g of edible flower

This study provides relevant data for the quality control of alternative foods used as dietary supplements containing UEFs, such as dandelion (*T. officinale*) and hibiscus (*H. sabdariffa*) from Brazil. Therefore, it is important to quantify these chemical elements due to their increased consumption by the population, either as part of the basic diet or as a source of nutrient supplementation. However, the scientific literature still lacks information on the application of the INFOGEST 2.0 in vitro protocol and MIP OES for evaluating the bioaccessibility of chemical species in edible flowers. This study directly contributes to achieving Sustainable Development Goals (SDGs), particularly 2 (Zero Hunger), 3 (Good Health and Well-Being), and 12 (Responsible Consumption and Production).

## Conclusion

Worldwide, consumption of new and unconventional foods is increasing. UEFs are a viable alternative for supplementation and can help address food insecurity. We investigated capsules containing dandelion and hibiscus powder from Brazil. A labeling assessment revealed significant nonconformities in all analyzed products. Proximate composition analysis revealed low levels of protein and lipids in both samples. Additionally, the hibiscus samples had a high ash content. Simulated in vitro gastrointestinal digestion tests, in conjunction with multielemental content analysis, revealed that these UEFs have promising mineral profiles. Although iron had high total levels, it was only 0.10% bioaccessible. Conversely, Mn and Ni exhibited high gastrointestinal release, accounting for 12.13% and 14.31% of the bioaccessible fractions, respectively. Furthermore, MIP OES use validated an efficient and accessible analytical method that aligns with sustainability principles.

Overall, the bioaccessibility results indicate that the nutritional value of these edible flowers hinges on the total elemental content and the amount released during digestion. The Mn bioaccessible fraction indicates that these products could contribute significantly to dietary Mn intake, pending future toxicological analyses. Therefore, assessing mineral bioaccessibility is essential for accurately estimating the nutritional impact of these products and ensuring safe consumption levels.

The main limitations of this study were the small sample size and the limited availability of dandelion and hibiscus powder products in oral capsule form in Brazil. Additionally, the study only established in vitro multielemental profiles based on the INFOGEST 2.0 protocol. Therefore, more testing is needed to confirm the safety and efficacy of these products as dietary supplements. Future steps could include calculations to predict toxicological risk parameters, membrane permeation assays, in vitro-in vivo pharmacokinetic correlations, and analysis of biological fluids from volunteers to determine bioavailability.

This study is the first to evaluate the bioaccessible trace element content of edible flowers using the INFOGEST 2.0 in vitro protocol and MIP OES to quantify microelements. Thus, this study advances our understanding of in vitro mineral bioaccessibility and nutritional and food safety assessments, as well as the development of UEF-based nutraceutical products. Future studies should sample a greater variety of edible flowers to improve the representativeness of nutritional and environmental assessments, particularly those available in capsule form in the Brazilian and global markets.

## Data Availability

No datasets were generated or analysed during the current study.
